# FGF21 attenuates pulmonary arterial hypertension via downregulation of miR‐130, which targets PPARγ

**DOI:** 10.1111/jcmm.17154

**Published:** 2022-01-06

**Authors:** Meibin Wang, Lihuang Su, Junwei Sun, Luqiong Cai, Xiuchun Li, Xiayan Zhu, Lanlan Song, Jingyin Li, Shuolan Tong, Qinlian He, Mengsi Cai, Lehe Yang, Yanfan Chen, Liangxing Wang, Xiaoying Huang

**Affiliations:** ^1^ Division of Pulmonary Medicine Key Laboratory of Heart and Lung The First Affiliated Hospital of Wenzhou Medical University Wenzhou Zhejiang China

**Keywords:** apoptosis, fibroblast growth factor 21, MiR‐130, proliferation, pulmonary arterial hypertension

## Abstract

The proliferation, migration and apoptotic resistance of pulmonary artery smooth muscle cells (PASMCs) are central to the progression of pulmonary arterial hypertension (PAH). Our previous study identified that fibroblast growth factor 21 (FGF21) regulates signalling pathway molecules, such as peroxisome proliferator‐activated receptor gamma (PPARγ), to play an important role in PAH treatment. However, the biological roles of miRNAs in these effects are not yet clear. In this study, using miRNA sequencing and real‐time PCR, we found that FGF21 treatment inhibited miR‐130 elevation in hypoxia‐induced PAH in vitro and in vivo. Dual luciferase reporter gene assays showed that miR‐130 directly negatively regulates PPARγ expression. Inhibition of miR‐130 expression suppressed abnormal proliferation, migration and apoptotic resistance in hypoxic PASMCs, and this effect was corrected upon PPARγ knockdown. Both the ameliorative effect of FGF21 on pulmonary vascular remodelling and the inhibitory effect on proliferation, migration and apoptotic resistance in PASMCs were observed following exogenous administration of miR‐130 agomir. In conclusion, this study revealed the protective effect and mechanism of FGF21 on PAH through regulation of the miR‐130/PPARγ axis, providing new ideas for the development of potential drugs for PAH based on FGF21.

## 1 INTRODUCTION

Hypoxia‐induced pulmonary arterial hypertension (PAH) is a chronic disease characterized by irreversible vascular remodelling and a progressive increase in pulmonary vascular resistance, ultimately leading to right ventricular failure and premature death.^1^, ^2^ Pulmonary artery smooth muscle cells (PASMCs) are a major component of pathological vascular remodelling, and their excessive proliferation, increased migration capacity and apoptotic resistance lead to pulmonary vascular obstruction, resulting in increased pulmonary artery pressure and right ventricular overload.^3^ Despite efforts to develop treatments to ameliorate symptoms and improve outcomes, mortality remains high.^4^ Therefore, there is an urgent need to identify new therapeutic targets.

Fibroblast growth factor 21 (FGF21) is an endocrine hormone with multiple biological functions that has demonstrated multiple biological effects.^5^, ^6^, ^7^ A growing body of data suggests that FGF21 is an important vasoprotective factor.^8^, ^9^ Our previous studies reported that FGF21 can inhibit hypoxia‐induced PASMC proliferation and migration by upregulating Peroxisome proliferator‐activated receptor gamma (PPARγ), promoting apoptosis and thereby downregulating inflammatory cytokine levels, improving collagen deposition in the lung and attenuating hypoxia‐induced PAH.^10^, ^11^ However, the exact mechanism of action remains unclear.

MicroRNAs (miRNAs) are highly conserved non‐coding RNAs that are expressed endogenously, and their dysregulation is an important factor in the development of cardiovascular injury, such as congestive heart failure and PAH.^12^, ^13^ Studies have shown that FGF21 ameliorates cardiovascular injury by regulating miRNA expression.^14^, ^15^ Bertero et al. reported that miR‐130 expression was increased in PAH, promoting the expression of phenotypes associated with pulmonary hypertension.^16^, ^17^, ^18^ Our high‐throughput sequencing and real‐time PCR (qPCR) results similarly showed that, compared to the hypoxic group, miR‐130 expression was significantly lower in the FGF21‐treated group. However, whether miR‐130 is involved in the signalling of the FGF21/PPARγ axis is unclear. This sparked our interest in this research.

Therefore, our study aimed to elucidate whether miR‐130 is involved in the alleviation of the FGF21/PPARγ axis in hypoxia‐induced PAH.

## 2 MATERIALS AND METHODS

### 2.1 Reagents

FGF21 was purchased from PeproTech. The miR‐130 agomir was obtained from RiboBio. The primary antibodies used in this study included PPARγ (Abcam, #ab178860), α‐SMA (Abcam, #ab5694), bcl‐2 (Abcam, #ab59348), proliferating cell nuclear antigen (PCNA; Cell Signalling Technology, #13110), β‐actin (Cell Signalling Technology, #4970), Bax (Cell Signalling Technology, #14796), caspase‐3 (Cell Signalling Technology, #9665), cleaved caspase‐3 (Cell Signalling Technology, #9665), apoptosis‐inducing factor (AIF; Cell Signalling Technology, #5318), cytochrome c (Cyt C; Cell Signalling Technology, #11940), cyclin‐dependent kinase 1 (CDK1; Affinity Biosciences, DF6024), cyclin D1 (Affinity Biosciences, AF0931) and COX IV (Affinity Biosciences, AF5468). The PPARγ agonist pioglitazone (Pio) was obtained from Selleck (Houston, TX, USA). Donkey anti‐mouse IgG H&L (Alexa Fluor 488; lot no. ab150105) antibodies were purchased from Abcam. Horseradish peroxidase (HRP)‐conjugated goat anti‐rabbit IgG antibody (lot no. BL003A) was purchased from Biosharp. Tissue Mitochondria Isolation Kit (#C3606) and Cell Mitochondria Isolation Kit (#C3601) were purchased from Beyotime. The One‐Step Terminal deoxynucleotidyl transferase dUTP nick end labelling (TUNEL) Apoptosis Assay Kit (#C1089) was purchased from Beyotime.

### 2.2 Cell culture and treatment

Pulmonary artery smooth muscle cells were isolated from male Sprague‐Dawley rats (weighing 180 ± 10 g) using well‐established methods, as previously described.^19^ PASMCs from passages four to six were used in all studies. PASMCs were divided into the following groups: 1. normoxia (Nor)+negative control (NC), hypoxia (Hyp)+NC, Hyp+miR‐130 inhibitor (100 nM), Hyp+miR‐130 inhibitor+siPPARγ (50 nM); 2. Nor+mimic control (MC), Hyp+MC, Hyp+MC+FGF21 (50 ng/ml), Hyp+FGF21+miR‐130 mimic (50 nM); 3. Nor+DMSO, Hyp+DMSO, Hyp+Pio (5 µm). The Nor group was cultured in a normal incubator with 21% O_2_, 74% N_2_ and 5% CO_2_ at 37 °C for 48 h. Hyp groups were maintained in an atmosphere of 5% O_2_, 90% N_2_ and 5% CO_2_ at 37 °C for 48 h. All groups were given treatment at the beginning of modelling.

### 2.3 Immunofluorescence

The cells were immobilized in 4% paraformaldehyde for 30 min and then permeabilized with 0.1% Triton X‐100. After blocking with 5% bovine serum albumin, the cells were incubated with anti‐PPARγ antibody (1:250, Abcam) or anti‐α‐smooth muscle actin antibody (1:100, Abcam) overnight at 4°C. The cells were then incubated with 1:1000 DyLight 488‐conjugated goat anti‐rabbit IgG (H+L; Abbkine Inc.), and nuclei were stained with 4,6‐diamidino‐2‐phenylindole (DAPI). Fluorescence images were captured using a fluorescence microscope (Leica DMi8).

### 2.4 Western blot analysis

Lung tissues were homogenized in cold radioimmunoprecipitation assay (RIPA) lysis buffer using an automatic homogenizer (FastPrep‐24 5G, MP Biomedicals) and lysed using an ultrasonic disruptor. The supernatants were collected after the homogenates were centrifuged (12,000 rpm, 4°C) for 30 min. PASMCs were lysed using RIPA according to standard procedures after washing with PBS three times. The protein content of each extract was measured using a BCA protein assay kit (Pierce Biotechnology). Subsequently, 10% sodium dodecyl sulphate‐polyacrylamide gel electrophoresis was used to separate proteins under denaturing conditions and then transferred to polyvinylidene fluoride (PVDF, Bio‐Rad) membranes. After blocking with 5% skim milk for 1 h, the membranes were washed with Tris‐buffered saline containing Tween 20. Then, the blots were incubated at 4°C overnight with rabbit primary antibodies against PPARγ (1:1000), PCNA (1:1000), CDK1 (1:1000), cyclin D1 (1:1000), Bax (1:1000), bcl‐2 (1:1000), cleaved caspase‐3 (1:1000), caspase‐3 (1:1000) and AIF (1:1000). They were then incubated with goat anti‐rabbit secondary antibody (1:10000) labelled with HRP at room temperature for 1 h. To detect Cyt C release, mitochondrial and cytosolic pellets were isolated using a Mitochondria Isolation Kit and immunoblotted with antibodies against Cyt C (1:1,000); COX IV served as the mitochondrial marker, and β‐actin was used as the cytosolic marker. After the pellets were triple washed with phosphate‐buffered saline, the protein bands were examined using the Bio‐Rad ChemiDoc MP imaging system and the quantification was performed using Image Lab software (Bio‐Rad).

### 2.5 Proliferation assays

The cells were seeded into 96‐well plates at a density of 1 × 10^4^ cells/well for 48 h. Then, 10 µl of cell counting kit‐8 (CCK8, Dojindo) reagent were added to each well, the cells were incubated at 37°C for 2 h and absorbance was measured at 450 nm. A 5‐ethynyl‐20‐deoxyuridine cell proliferation assay kit (Abcam) was used to analyse cell proliferation.

### 2.6 Detection of apoptosis

A One‐Step TUNEL Apoptosis Assay Kit was used to detect apoptosis. The cells were seeded into 24‐well plates at a density of 2 × 10^4^ cells/well for 48 h. After washing with PBS three times, the cells were immobilized in 4% paraformaldehyde for 30 min and permeabilized with 0.1% Triton X‐100. The cells were then incubated with the TUNEL reaction mixture, as instructed. Fluorescence images were captured using a fluorescence microscope (Leica DMi8).

### 2.7 Transwell migration chamber assay

A 24‐well Transwell system (8 µm; Corning Incorporated, NY, USA) was used for the transwell assay. DMEM containing 5% FBS was added to the lower chamber of the transwell plates with the experimental treatments in each group. PASMCs were suspended in DMEM and seeded into the upper chambers at a density of 2 × 10^4^ cells/well. After incubation for 24 h under normoxic or hypoxic conditions, non‐migrated cells in the upper chamber were scraped. The migrated cells were fixed with 4% paraformaldehyde and stained with crystal violet. A microscope (Leica DMi8) was used to count the migrated cells.

### 2.8 Wound‐healing assay

The cells were seeded into six‐well plates at a density of 2 × 10^5^ cells/well for 24 h. The cells were then serum‐deprived for 24 h. Afterwards, the cell monolayers were scratched with 200‐µl pipette tips in the centre of the well and incubated with the experimental treatments for 48 h. ImageJ (NIH, USA) was used to determine the level of wound‐healing cover.

### 2.9 Animal models

Male C57BL/6 mice (8–12 weeks old, 20–25 g) were purchased from Vital River Laboratory Animal Technology. The experimental scheme and the animal housing were approved by the Animal Ethics Committee of Wenzhou Medical University. The mice were raised in a humidity range of 55%–65% and 20–24°C. To verify the function of FGF21 and the change in miR‐130 expression, mice were divided into three groups randomly: normoxia (Nor, saline‐treated) group, hypoxia (Hyp, saline‐treated) group and hypoxia+FGF21 (Hyp+FGF21, FGF21, 0.2 mg/kg i.p.) group. To investigate whether FGF21 attenuated hypoxia‐induced PAH by inhibiting the negative regulatory effects of miR‐130 on PPARγ, mice were divided into five groups randomly: normoxia (Nor, saline‐treated) group, hypoxia (Hyp, saline‐treated) group, hypoxia+FGF21 (Hyp+FGF21, FGF21, 0.2 mg/kg i.p.) group, hypoxia+FGF21+negative control agomir (Hyp+FGF21+NC, FGF21, 0.2 mg/kg i.p.; negative control agomir, 20 nmol) group and hypoxia+FGF21+miR‐130 agomir (Hyp+FGF21+Agomir, FGF21, 0.2 mg/kg i.p.; miR‐130 agomir, 20 nmol) group. All hypoxia groups were raised in a normobaric chamber with 10% O_2_ for 21 days, and the normoxia group was housed in ambient air. All FGF21 treatment groups received injections once a day for 21 days, beginning on the first day of modelling. Mice were injected with miR‐130 agomir every 3 days from modelling for 21 days.

### 2.10 Ethics approval

All animal procedures conformed to the guidelines from Directive 2010/63/EU of the European Parliament on the protection of animals used for scientific purposes or the current NIH guidelines and were approved by the Animal Ethics Committee of Wenzhou Medical University. All animals were handled with care and euthanized humanely during the study.

### 2.11 Echocardiography

Transthoracic echocardiography was performed using a Visual Sonics Vevo 3100 ultrasound machine. After anaesthetization with continuous isoflurane inhalation (1.5%–3.0%), mice were placed on a heated pad in a supine position, and the fur on their chest was removed with a chemical hair remover. The pulmonary artery acceleration time (PAAT) and velocity time integral (VTI) were obtained from the modified parasternal long‐axis view using the pulsed Doppler mode.

### 2.12 Measurement of haemodynamic and right ventricular hypertrophy

The mice were anaesthetized with 20% urethane (1 ml/100 g, i.p.), and catheters were inserted into the right ventricular (RV) and left carotid arteries.^10^ Right ventricular systolic pressure (RVSP) was recorded using pressure transducers (PowerLab 8/35 Multichannel Biological Signal Recording System, AD Instruments, AUS). The animals were sacrificed for further examination. The RV was dissected from the septum (S) and left ventricle (LV), and RV hypertrophy was defined as the ratio of RV to (LV+S) weight.

### 2.13 Measurement of pulmonary arterial remodelling and arterial collagen deposition

Pulmonary arterial remodelling was assessed using the method described by Cai et al.^10^ Briefly, connective tissue around the lung was removed, and the lung tissue was fixed in 4% paraformaldehyde for 24 h. Then, the lung tissue was cut into 5‐µm thick slices after being embedded in paraffin. Sections were then stained with haematoxylin‐eosin (H&E), and an optical microscope was used to evaluate the structure of the pulmonary arteries with external diameters of 25–100 µm. Image‐Pro Plus 6.0, (Media Cybernetics) was used to analyse the ratio of the wall thickness (WT) to the total thickness (TT, WT/TT) and the pulmonary artery wall area (WA) to the total area (TA, WA/TA). To assess arterial collagen deposition, sections were stained with Masson staining. An optical microscope was used to observe arteries with external diameters of 25–100 µm. The collagen fibres were blue, and the erythrocytes, cytoplasm and muscle fibres were stained red. The ratios of the collagen fibre area to the pulmonary artery WA were analysed using ImageJ (NIH).

### 2.14 sRNA sequencing

Total RNA was extracted using the TRIzol reagent (Invitrogen). The quantity and integrity of the products were evaluated using Qubit^®^2.0, (Life Technologies) and Agilent 2200 TapeStation (Agilent Technologies). For sRNA sequencing, 1 µg of the mixed total RNA was used for sRNA library construction. The sRNA libraries were constructed using the NEBNext^®^ Multiplex Small RNA Library Prep Set for Illumina (NEB) and sequenced on a HiSeq 2500 (Illumina) platform at RIBOBIO (Guangzhou, China).

### 2.15 MiRNA target prediction

Identification of the putative miRNA target was performed using miRNA target analysis tools at miRDB (http://mirdb.org/miRDB/), TargetScan (http://www.targetscan.org/) and starBase (http://starbase.sysu.edu.cn/).

### 2.16 qRT‐PCR

MiRNA was extracted from PASMCs using a SanPrep Column microRNA Extraction Kit (Sangon Biotech, Shanghai, China) following the manufacturer's instructions. RNA was quantified using an ultraviolet spectrophotometer, and only samples with an OD260/OD280 ratio greater than 1.8, were used for experiments. The cDNA was synthesized using a miRNA First‐Strand cDNA Synthesis Kit (stem‐loop method) from Sangon Biotech (Shanghai, China). Real‐time PCR was performed using a MicroRNA qPCR Kit (SYBR Green; Sangon Biotech, Shanghai, China) according to the manufacturer's protocol. U6 small nuclear RNA (snRNA) was selected as an endogenous control for miR‐130, and the 2^−ΔΔCt^ method was used to calculate the relative expression of the detected gene. The primer sequences used for miRNA and mRNA analysis are shown in Table S1 and S2.

### 2.17 Small interfering RNA (siRNA) design and transfection

For PPARγ knockdown, PASMCs were transfected with small interfering RNA (siRNA) synthesized by RiboBio (Guangzhou, China). Non‐targeted control siRNA was used as a negative control. The sense sequences for the siRNA, inhibitor and mimic are as follows: siPPARγ: 5’‐CCGCCTTATTATTCTGAAA‐3’, inhibitor: 5’‐AUGCCCUUUCAUCAUUGCACUG‐3’ and mimic 5’‐CAGUGCAAUGAUGAAAGGGCAU‐3’. A Ribo FECT™ CP transfection kit was used for siRNA, inhibitor, or mimic transfection. After transfection, the cells were used for further experiments.

### 2.18 Dual‐luciferase reporter assay

The 3’UTR of PPARγ mRNA with a putative/mutant miR‐130‐binding site was cloned into the pmiR‐RB‐ReportTM vector (RiboBio). Firefly luciferase was selected as a reporter, and Renilla luciferase was selected as a control. PASMCs were transfected with 50 nM miR‐130 mimic and 100 ng pmirGLO vector using the Attractene Transfection Reagent (Qiagen). Luciferase activity was measured using a Dual‐Luciferase^®^ Reporter Assay System (E1910, Promega) after incubation for 48 h.

### 2.19 Drawing software

Statistical histograms were drawn using GraphPad Prism 7.0 (Graph Pad Software Inc.), and the overall layout was constructed using Adobe Illustrator CS6 (Adobe Illustrator Software Inc).

### 2.20 Statistical analysis

Statistical analyses were performed using GraphPad Prism 7.0 (Graph Pad Software Inc.). The results are presented as the means ±standard deviation (SD). Student's *t*‐test was used to compare the two groups. Multiple comparisons were performed using one‐way ANOVA. Statistical significance was set at *p* < 0.05.

## 3 RESULTS

### 3.1 FGF21 can alleviate hypoxia‐induced pulmonary arterial remodelling and reverse hypoxia‐induced PAH

**FIGURE 1:**
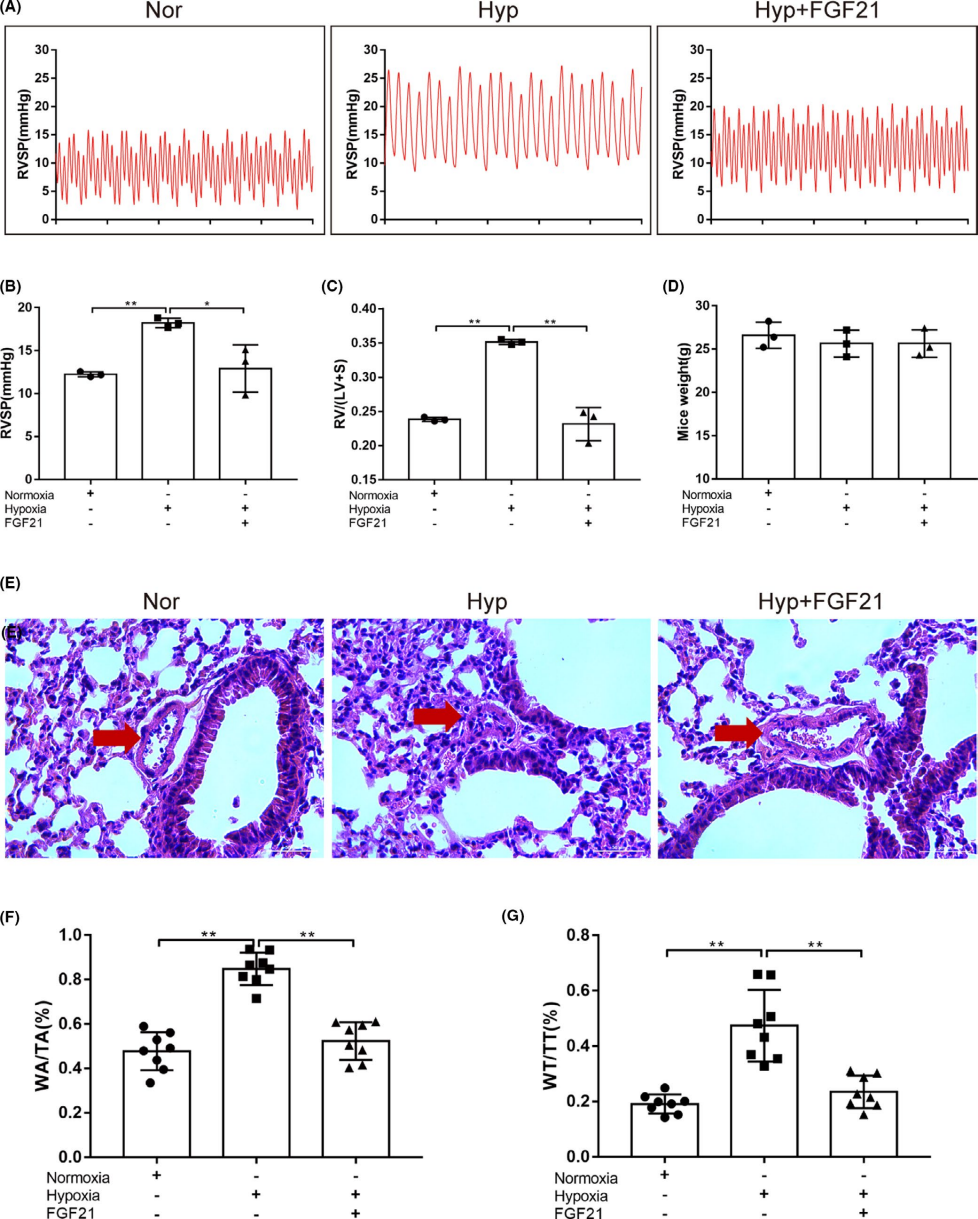
FGF21 can alleviate hypoxia‐induced pulmonary arterial remodelling and reverse hypoxia‐induced PAH. (A) Representative images of right ventricular systolic pressure (RVSP) waves of normoxia (Nor), hypoxia (Hyp) and hypoxia+fibroblast growth factor 21 (FGF21, Hyp+FGF21) groups. (B) RVSP of Nor, Hyp and Hyp+FGF21 groups (*n* = 3). (C) Right ventricular (RV) hypertrophy shown as the RV/(LV+S) ratio (*n* = 3). (D) Mice weight in each group (*n* = 3). (E) H&E staining was used to evaluate pulmonary vascular remodelling. Representative photomicrographs showing hypoxia‐induced remodelling in the pulmonary arteries of mice exposed to hypoxia (10% O_2_) or ambient oxygen levels (21% O_2_) for 3 weeks (×400; scale bar indicates 50 µm), red arrow indicates the pulmonary artery. (F) The ratios of the pulmonary artery wall area (WA) to the total area (TA, WA/TA) and (G) the wall thickness (WT) to the total thickness (TT, WT/TT) for each group (*n* = 3). Data are presented as the mean ±SD. *p < 0.05, **p < 0.01

As shown in Figure 1A–C, RVSP and RV/(LV+S) were significantly increased in the Hyp group compared with the Nor group (*p* < 0.01), and FGF21 significantly inhibited the hypoxia‐induced increase in pulmonary artery pressure and ventricular remodelling level (*p* < 0.05, *p* < 0.01). There was no significant difference in heart rate and body weight between the groups (Figure 1D). Moreover, sections stained with H&E showed that hypoxia‐induced pulmonary arterial remodelling was alleviated in the Hyp+FGF21 group, compared with the Hyp group and WA/TA (%) and WT/TT (%) showed the same trend (*p* < 0.01; Figure 1E,G). In conclusion, consistent with previous findings,^10^, ^11^ FGF21 can improve hypoxia‐induced pulmonary remodelling and alleviate hypoxia‐induced PH.

### 3.2 The expression of miR‐130 increases in PAH mice, and FGF21 can significantly downregulate miR‐130 expression

**FIGURE 2:**
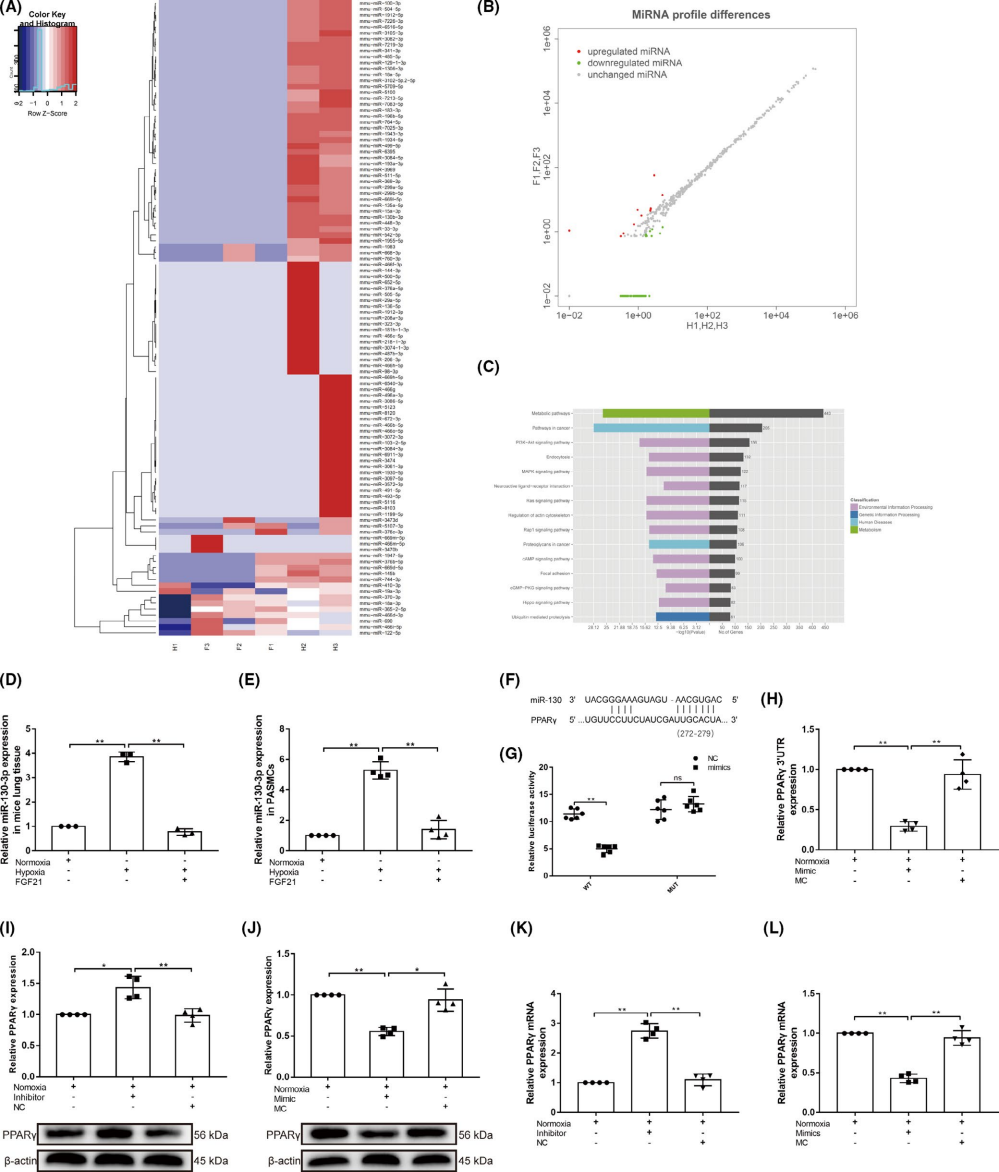
FGF21 can significantly downregulate miR‐130 expression in PAH mice, and miR‐130 can directly target PPARγ and inhibit its expression. (A) Heatmap diagram revealing all differentially expressed microRNAs (miRNAs) in the Hyp and Hyp+FGF21 groups compared in lung tissues, ranging from the most upregulated (red) to the most downregulated (blue). (B) Scatter plot demonstrating differential miRNA expression in the two groups. Red = miRNAs with higher expression, green = miRNAs with lower expression and black = miRNAs with equal expression. (C) The top 15 pathways of the target genes of dysregulated miRNA were identified using KEGG analysis according to the number of enriched genes. (D) qRT‐PCR was used to detect miRNA expression of miR‐130 in the Nor, Hyp and Hyp+FGF21 groups mice (*n* = 3) and (E) pulmonary arterial smooth muscle cells (PASMCs; *n* = 4). The miRNA level is normalized to U6 by the 2^−ΔCt^ method before comparative analysis. (F) Potential targeted binding between miR‐130 and peroxisome proliferator‐activated receptor gamma (PPARγ) was predicted by using miRDB, TargetScan and starBase web tools. (G) The identified complementary binding sites were validated by performing dual‐luciferase reporter (*n* = 6). (H) qRT‐PCR was used to detect PPARγ 3ʹ‐UTR expression. The mRNA level is normalized to β‐actin by the 2^−ΔCt^ method before comparative analysis (*n* = 4). (I and J) Western blotting for PPARγ expression in PASMCs transfected with miR‐130 mimic and miR‐130 inhibitor (*n* = 4), β‐actin was used as a loading control. (K and L) qRT‐PCR was used to detect mRNA expression of PPARγ in PASMCs transfected with miR‐130 mimic and miR‐130 inhibitor (*n* = 4). The mRNA level is normalized to β‐actin by the 2^−ΔCt^ method before comparative analysis. Data are presented as the mean ± SD. *p < 0.05, **p < 0.01

To explore the internal mechanism of FGF21 in the treatment of PAH, lung samples from the Hyp and Hyp+FGF21 groups were collected for miRNA high‐throughput sequencing. We found a significant reduction in miRNAs after FGF21 intervention and identified the top 15 KEGG pathways in the lung (Figure 2A–C). PCR was used to verify the screened differentially expressed miRNAs, and it was found that miR‐130 expression in the hypoxia group was significantly higher than in the normoxia group, and FGF21 treatment significantly downregulated its level (*p* < 0.01; Figure 2D,E). This evidence suggests that FGF21 may alleviate PAH by downregulating the expression of miR‐130.

### 3.3 MiR‐130 can directly target PPARγ and inhibit its expression

Based on the bioinformatics database, miR‐130 was predicted to target the PPARγ gene (Figure 2F). This correlated with the expectations. Accumulating evidence suggests that PPARγ reverses pulmonary artery remodelling, PAH and RV hypertrophy (RVH).^20^, ^21^, ^22^ Our study also demonstrated that PPARγ inhibits hypoxia‐induced PASMC proliferation and migration while promoting apoptosis (*p* < 0.05/*p* < 0.01; Figure S1–S3). To further confirm this hypothesis, we performed a luciferase reporter gene assay to test the direct interaction between PPARγ and miR‐130. The results showed that the fluorescence activity of miR‐130 mimic transfection was significantly reduced compared with control miRNA transfection (*p* < 0.01; Figure 2G); however, no effect was observed when miR‐130 mimic was co‐transfected with the mutant PPARγ 3'‐UTR. Meanwhile, PCR showed that the expression level of PPARγ 3'UTR was significantly decreased (*p* < 0.01) by transfection of miR‐130 mimic (Figure 2H). The above results suggest a strong interaction between miR‐130 and the PPARγ 3'‐UTR. PCR and western blotting analysis further confirmed that miR‐130 could inhibit the mRNA and protein expression levels of PPARγ [miR‐130 inhibitor efficiency was verified using PCR (Figure S4A; *p* < 0.01)]. Conversely, inhibition of miR‐130 reversed PPARγ expression in PASMCs (*p* < 0.05/*p* < 0.01; Figure 2I–L). These results suggest that miR‐130 specifically targets and inhibits PPARγ expression.

### 3.4 MiR‐130 promotes hypoxia‐induced PASMC proliferation and migration and inhibits apoptosis by regulating PPARγ expression

**FIGURE 3:**
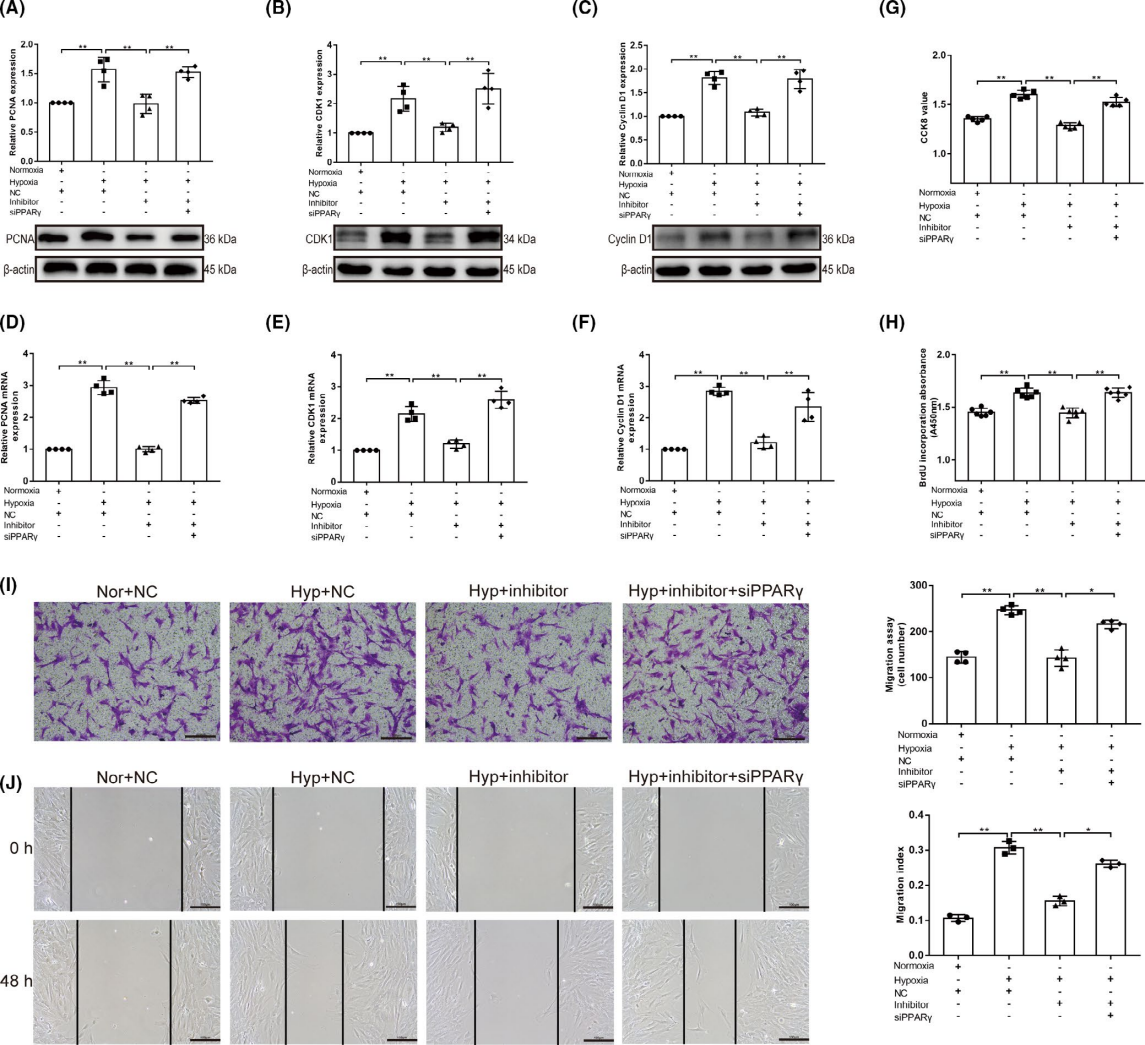
MiR‐130 increases hypoxia‐induced PASMC proliferation and migration by regulating PPARγ expression. (A–C) Western blotting for proliferating cell nuclear antigen (PCNA), cyclin‐dependent kinase 1 (CDK1) and cyclin D1 in Nor, Hyp, Hyp+miR‐130 inhibitor and Hyp+miR‐130 inhibitor+siPPARγ groups, β‐actin was used as a loading control (*n* = 4). (D–F) qRT‐PCR was used to detect mRNA expression of PCNA, CDK1 and cyclin D1 in each group. The mRNA level is normalized to β‐actin by the 2^−ΔCt^ method before comparative analysis (*n* = 4). (g) CCK‐8 assay (*n* = 5) and (H) BrdU incorporation study (*n* = 6) were used to evaluate cell viability. (I) Cell migration was determined by Transwell assays at 24 h (*n* = 4; ×100; scale bars indicate 200 µm). (J) Cell migration was determined by wound‐healing assays at 0 and 48 h (*n* = 3) (×200; scale bars indicate 100 µm). Data are presented as the mean ± SD. *p < 0.05, **p < 0.01

**FIGURE 4:**
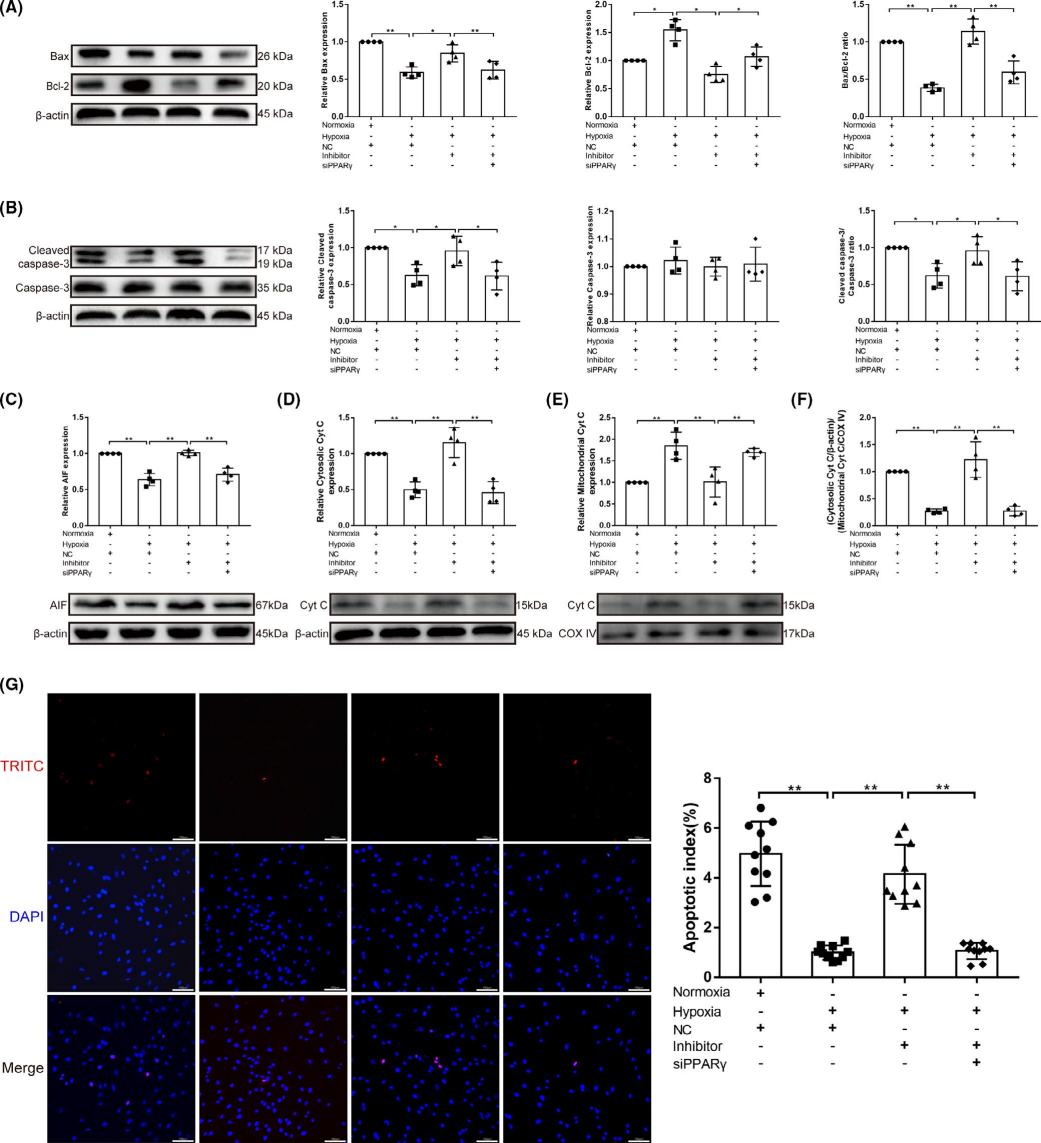
MiR‐130 inhibits hypoxia‐induced PASMC apoptosis by regulating PPARγ expression. (A–C) Western blotting for Bax, Bcl‐2, cleaved caspase‐3, caspase‐3 and apoptosis‐inducing factor (AIF) expression in PASMCs in Nor, Hyp, Hyp+miR‐130 inhibitor and Hyp+miR‐130 inhibitor+siPPARγ groups, β‐actin was used as a loading control (*n* = 4). (D‐F) The expression levels of cytochrome c (Cyt C) in mitochondrial and cytosol pellets in PASMCs were examined by western blotting with antibodies against Cyt C with COX IV as a mitochondria marker and β‐actin as the internal control (*n* = 4). (G) The apoptosis index of PASMCs in each group was measured by TUNEL assay (*n* = 10) (×200; scale bars indicate 100 µm) and is shown as the ratio of TUNEL positive cells (red) to total cells (blue). Data are presented as the mean ± SD. *p < 0.05, **p < 0.01

The functional effects of miR‐130 on PPARγ were also studied. In PASMCs, siRNA‐knockdown efficiency was first analysed using a western blot assay, and the siRNA with the highest knockdown efficiency was selected (*p* < 0.05; Figure S5A). In PPARγ‐knockdown cells, the miR‐130 inhibitor failed to inhibit hypoxia‐induced PASMC proliferation (*p* < 0.01; Figure 3A–H). Additionally, a significantly higher migration ratio was observed in hypoxic PASMCs than in control cells (*p* < 0.01). However, migration ability was decreased by the miR‐130 inhibitor (*p* < 0.01), which was reversed by co‐transfection with siPPARγ (*p* < 0.05; Figure 3I,J). In conclusion, miR‐130 can increase hypoxia‐induced PASMC proliferation and migration by regulating PPARγ expression. Furthermore, we explored the role of miR‐130 in hypoxia‐induced PASMC apoptosis. Mitochondrial apoptosis‐related protein expression was measured using western blotting. Hypoxia downregulated the Bax/Bcl‐2 ratio, cleaved caspase‐3/caspase‐3 ratio and AIF expression (*p* < 0.01, *p* < 0.05), which was reversed by the miR‐130 inhibitor (*p* < 0.01, *p* < 0.05; Figure 4A–C). The release of Cyt C from the mitochondria to the cytoplasm was significantly inhibited in the hypoxia group, compared with the normoxia group (*p* < 0.01), which was reversed by the miR‐130 inhibitor (*p*<0.01; Figure 4D–F). Notably, the miR‐130 inhibitor‐induced increase in PASMC apoptosis was inhibited by PPARγ knockdown (*p* < 0.01, *p* < 0.05). Moreover, the results of the TUNEL assay showed the same changes (*p* < 0.01; Figure 4G). These results indicate that miR‐130 can inhibit apoptosis by regulating PPARγ expression.

### 3.5 FGF21 reduces proliferation and migration and enhances apoptosis of hypoxia‐induced PASMCs by inhibiting the negative regulatory effects of miR‐130 on PPARγ

**FIGURE 5:**
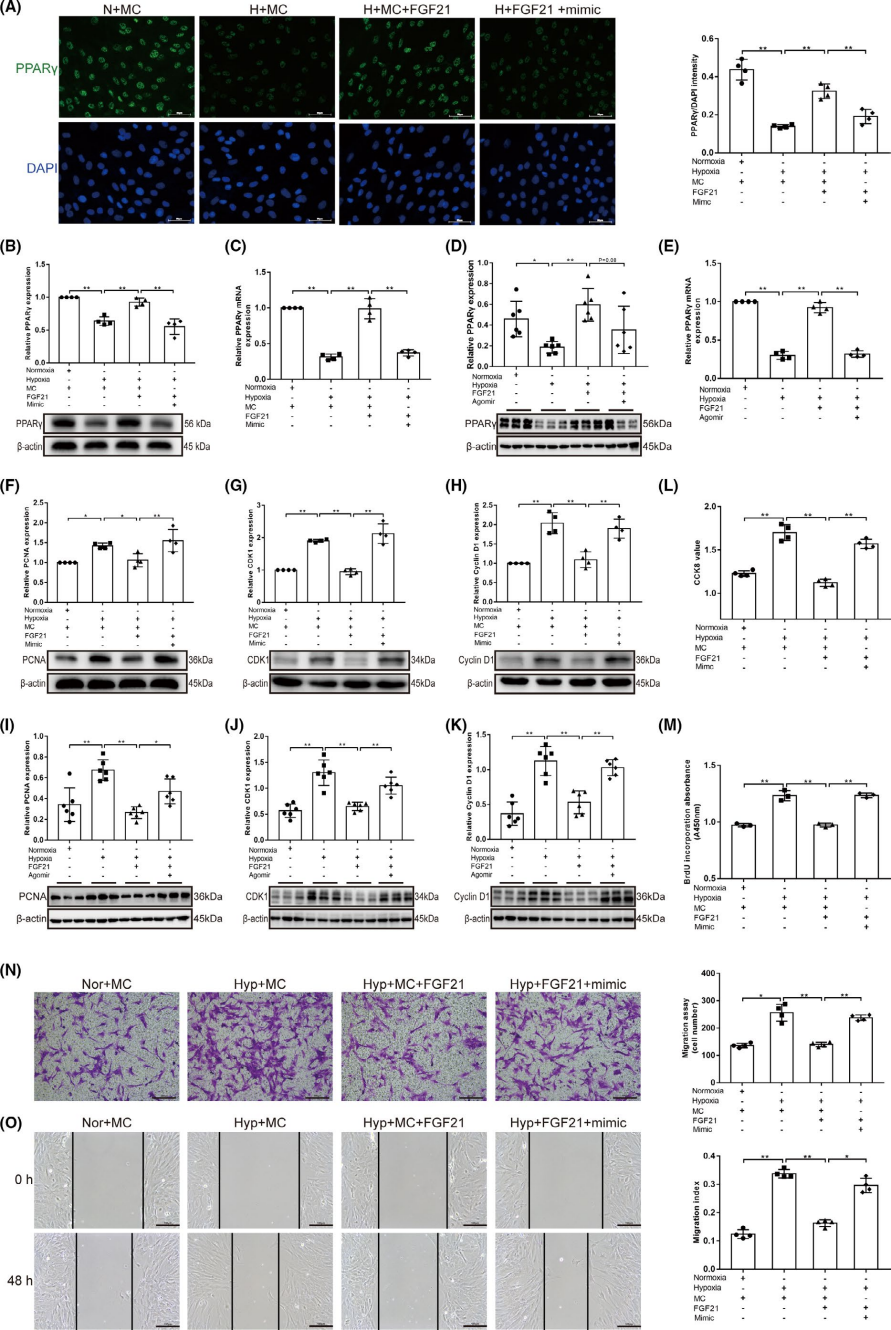
FGF21 reduces the proliferation and migration of hypoxia‐induced PASMCs by inhibiting the negative regulatory effects of miR‐130 on PPARγ. (A) Immunofluorescent staining for PPARγ (green) and DAPI (nuclear DNA; in blue). The histogram represents the quantification of the ratio of PPARγ to DAPI intensity (*n* = 4) (×400; scale bars indicate 50 µm). (B) Western blotting for PPARγ in Nor+mimic control (MC), Hyp+MC, Hyp+MC+FGF21, Hyp+FGF21+miR‐130 mimic groups, β‐actin was used as a loading control (*n* = 4). (C) qRT‐PCR was used to detect the expression of PPARγ in vitro. The mRNA level is normalized to β‐actin by the 2^−ΔCt^ method before comparative analysis (*n* = 4). (D) Western blotting for PPARγ in Nor, Hyp, Hyp+FGF21, Hyp+FGF21+NC and Hyp+FGF21+Agomir groups mice, β‐actin was used as a loading control (*n* = 6). (E) qRT‐PCR was used to detect the expression of PPARγ in *vivo*. The mRNA level is normalized to β‐actin by the 2^−ΔCt^ method before comparative analysis (*n* = 4). (F–H) Western blotting for PCNA, CDK1 and cyclin D1 in Nor+MC, Hyp+MC, Hyp+MC+FGF21, Hyp+FGF21+miR‐130 mimic groups, β‐actin was used as a loading control (*n* = 4). (I–K) Western blotting for PCNA, CDK1 and cyclin D1 in lung homogenates of mice, β‐actin was used as a loading control (*n* = 6). (L) CCK‐8 assay (*n* = 4) and (M) BrdU incorporation study (*n* = 3) were used to evaluate cell viability. (N) Cell migration was determined by Transwell assays at 24 h (*n* = 4) (×100; scale bars indicate 200 µm). (0) Cell migration was determined by wound‐healing assays at 0 and 48 h (*n* = 4) (×200; scale bars indicate 100 µm). Data are presented as the mean ±SD. *p < 0.05, **p < 0.01

**FIGURE 6:**
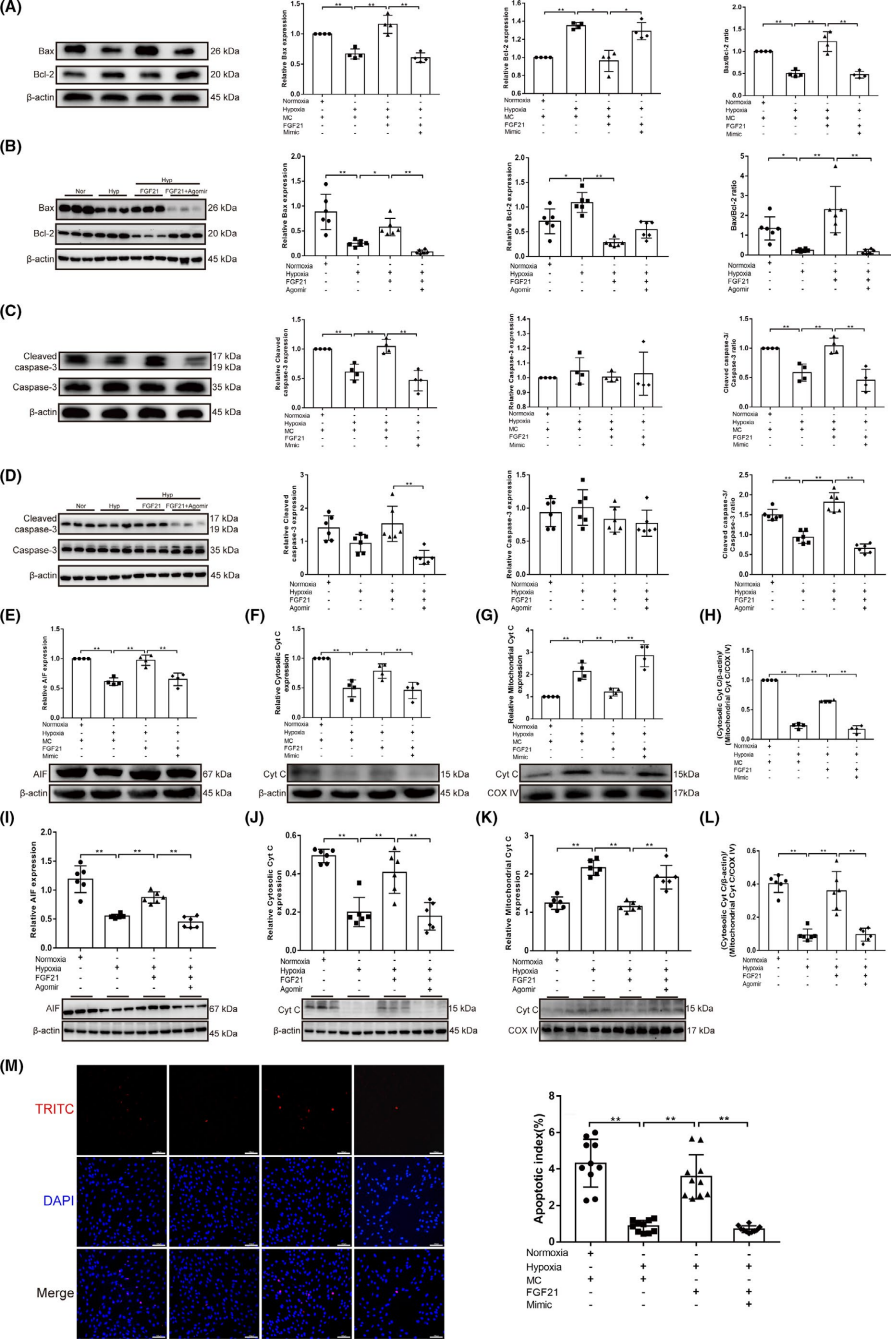
FGF21 enhances apoptosis in hypoxia‐induced PASMCs by inhibiting the negative regulatory effects of miR‐130 on PPARγ. (A, C, E) Western blotting for Bax, Bcl‐2, cleaved caspase‐3, caspase‐3, AIF expression in PASMCs in Nor+MC, Hyp+MC, Hyp+MC+FGF21, Hyp+FGF21+miR‐130 mimic groups, β‐actin was used as a loading control (*n* = 4). (B, D, I) Western blotting for Bax, Bcl‐2, cleaved caspase‐3, caspase‐3 and AIF in lung homogenates of mice (*n* = 6). (F–H) The expression levels of Cyt C in mitochondrial and cytosol pellets in PASMCs were examined by western blotting with antibodies against Cyt C with COX IV as a mitochondria marker and β‐actin as the internal control (*n* = 4). (J–L) The expression levels of Cyt C in mitochondrial and cytosol pellets in lung homogenates of mice were examined by western blotting with antibodies against Cyt C with COX IV as a mitochondria marker and β‐actin as the internal control (*n* = 6). (M) The apoptosis index of PASMCs in each group was measured by TUNEL assay (*n* = 10) (×200; scale bars indicate 100 µm) and is shown as the ratio of TUNEL positive cells (red) to total cells (blue). Data are presented as the mean ±SD. *p < 0.05, **p < 0.01

To confirm whether FGF21 regulates hypoxia‐induced PASMC proliferation and migration by inhibiting the negative regulatory effect of miR‐130 on PPARγ, we treated the cells with FGF21 and miR‐130 mimic and found that FGF21 upregulation of PPARγ was abolished by miR‐130 mimic under hypoxic conditions (*p* < 0.01; Figure 5A–C and Figure S6A). In addition, western blotting analysis and PCR also showed that FGF21 increased the expression of PPARγ in the Hyp+FGF21 group compared with the control group *in vivo* (*p* < 0.01), and administration of miR‐130 reversed this change (*p* < 0.01/*p* < 0.05; Figure 5D,E). The expression of PCNA, CDK1 and cyclin D1 was upregulated by miR‐130 in FGF21‐treated hypoxic PASMCs and mice (*p* < 0.01/*p* < 0.05; Figure 5F–K and Figure S6B–G), which indicated that the proliferation of hypoxic PASMCs inhibited by FGF21 could be reversed by miR‐130. The *in vitro* findings, as well as the CCK8 and BrdU incorporation findings, were also in line with the aforementioned results (*p*<0.01; Figure 5L,M). Transwell and wound‐healing assays showed that FGF21 inhibited hypoxia‐induced PASMC migration (*p* < 0.01; Figure 5N,O). However, this effect was attenuated by transfection with the miR‐130 mimic (*p* < 0.01/*p* < 0.05). In conclusion, our results indicate that FGF21 inhibits hypoxia‐induced PASMC migration and proliferation by downregulating miR‐130, which targets PPARγ. To further clarify whether FGF21 can affect PASMC apoptosis by reversing miR‐130 targeting PPARγ, the expression of apoptosis‐related proteins was measured using western blotting. Upon FGF21 treatment, the Bax/Bcl‐2 ratio was increased in hypoxic PASMCs and mice (*p* < 0.01), which was completely abolished by the addition of miR‐130 (*p* < 0.01; Figure 6A,B). The cleaved caspase‐3/caspase‐3 ratio, AIF expression and the release of Cyt C from the mitochondria to the cytoplasm showed the same trend both in vivo and in vitro (*p* < 0.01/*p* < 0.05; Figure 6C–L). The TUNEL assay also showed the same results (*p* < 0.01; Figure 6M). These results indicate that FGF21 enhances apoptosis in hypoxia‐induced PASMCs by inhibiting the negative regulatory effects of miR‐130 on PPARγ.

### 3.6 FGF21 attenuates chronic hypoxia‐induced PAH by inhibiting the negative regulatory effects of miR‐130 on PPARγ

**FIGURE 7:**
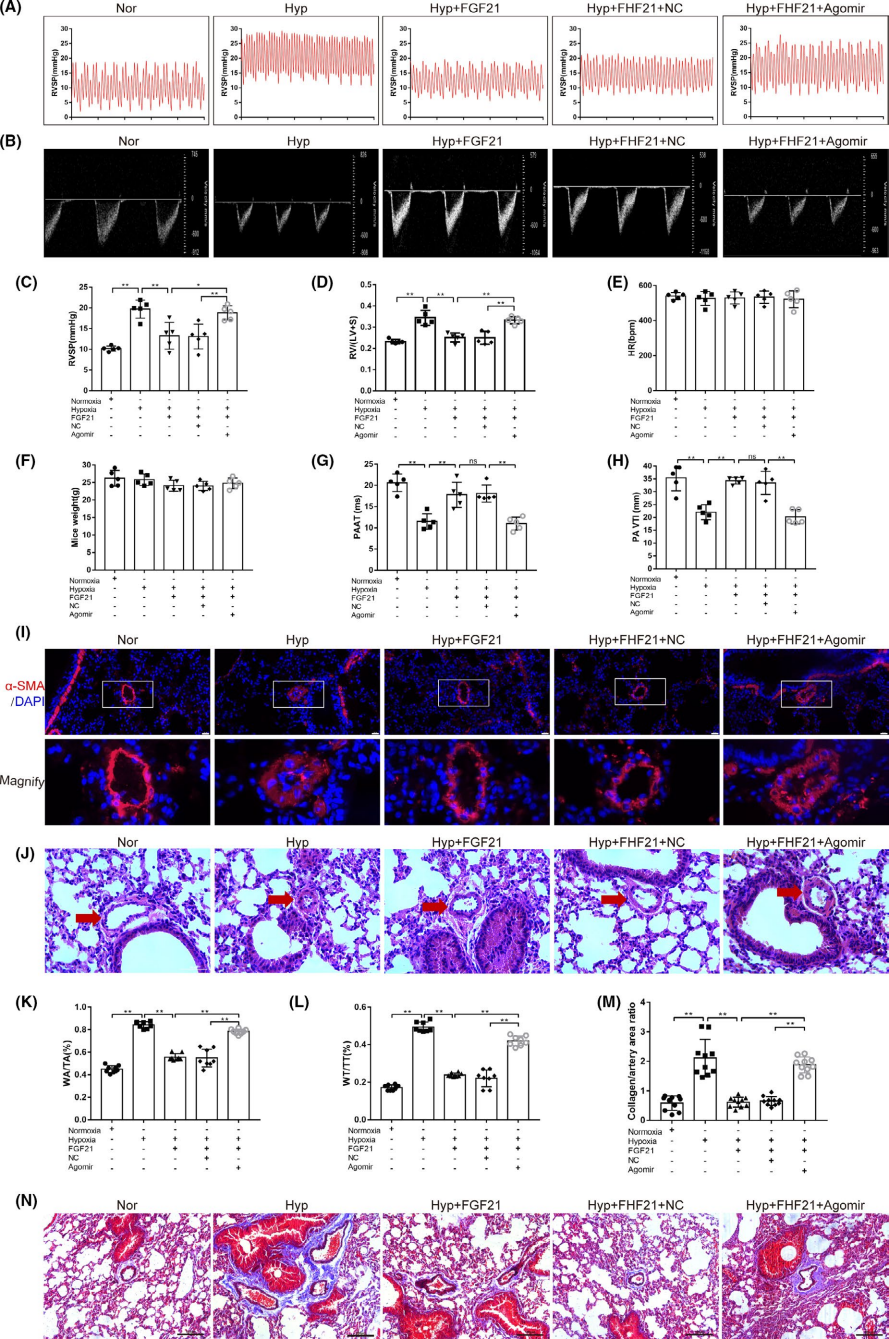
FGF21 attenuates chronic hypoxia‐induced PAH by inhibiting the negative regulatory effects of miR‐130 on PPARγ. (A) Representative pictures of right ventricular systolic pressure (RVSP) waves in Nor, Hyp, Hyp+FGF21, Hyp+FGF21+NC and Hyp+FGF21+Agomir groups (*n* = 5). All hypoxia groups were raised in a normobaric chamber with 10% O_2_ for 21 days. (B) Representative images of transthoracic echocardiography. (C) Measurement of RVSP and (D) RV/(LV+S) values in each group (*n* = 5). (E) Heart rate (HR, bpm; *n* = 5). (F) Mice weight in each group (*n* = 5). (G) Quantification of pulmonary artery acceleration time (PAAT) and (H) velocity time integral (VTI; *n* = 5). (I) Representative images of alpha‐smooth muscle actin (α‐SMA; in red) and DAPI (nuclear DNA; in blue) immunostaining of the distal pulmonary arteries in each group (×200, scale bars indicate 50 µm). (J) H&E staining was used to evaluate pulmonary vascular remodelling. Representative photomicrographs showing hypoxia‐induced remodelling in the pulmonary arteries of mice exposed to hypoxia (10% O_2_) or ambient oxygen levels (21% O_2_) for 3 weeks (×400; scale bars indicate 50 µm). (K) Quantification of WA/TA (%) and (L) WT/TT (%) ratios in each group (*n* = 8). (M) The degree of collagen deposition (blue) in each group was evaluated microscopically by Masson staining and is shown as the ratio of the collagen fibre area (blue) to the pulmonary artery wall area (red; *n* = 10). (N) Representative photomicrographs showing hypoxia‐induced collagen deposition (blue) around the pulmonary arteries (×200; scale bars indicate 100 µm). Data are presented as the mean ±SD. *p < 0.05, **p < 0.01

To further verify that FGF21 regulates PPARγ expression by regulating miR‐130, mice were exposed to hypoxia for three weeks and treated with FGF21 or miR‐130 agomir [efficiency of exogenous miR‐130 agomir administration in *vivo* was verified using PCR (Figure S7; *p* < 0.01)]. As shown in Figure 7A,C, FGF21 could significantly downregulate RVSP in the Hyp+FGF21 group mice, compared with the Hyp group (*p* < 0.01), and this decrease was reversed after exogenous miR‐130 agomir administration in the Hyp+FGF21+Agomir group (*p* < 0.05). Moreover, RV/(VL+S) was lower in the Hyp+FGF21 group than in the Hyp group (*p* < 0.01), and exogenous miR‐130 agomir administration increased the level of RV/(VL+S) in FGF21‐treated mice (*p *< 0.01; Figure 7D). There was no significant difference in heart rate and body weight between the groups (Figure 7E,F). Transthoracic echocardiography showed that FGF21 could inhibit hypoxia‐induced downregulation of PAAT and PAVTI, and miR‐130 agomir reversed these changes (*p* < 0.01; Figure 7B,G,H). Immunofluorescence results showed that PASMC proliferation was obviously stimulated under hypoxic conditions, while FGF21 prominently reduced this increased proliferation, and this effect could be weakened by exogenous miR‐130 (Figure 7I). Moreover, H&E‐stained sections showed that hypoxia‐induced pulmonary arterial remodelling was alleviated in the Hyp+FGF21 group, compared with the Hyp group, which could be reversed by exogenous miR‐130 agomir administration (Figure 7J). As expected, FGF21 reversed the hypoxia‐induced increase in the pulmonary artery WT/TT ratio (%) and WA/TA ratio (%), while these effects were substantially alleviated in miR‐130‐treated mice (*p* < 0.01; Figure 7K,L), further confirming that FGF21 could reverse hypoxia‐induced PASMC hyperplasia by inhibiting miR‐130 expression. Additionally, Masson staining showed that pulmonary artery collagen content was higher in the Hyp group than in the Nor group, which was reversed by FGF21 treatment (*p* < 0.01). As expected, exogenous miR‐130 agomir weakened the protective effect of FGF21 (*p* < 0.01; Figure 7M,N). Taken together, our results show that FGF21 can improve pulmonary vascular remodelling and collagen deposition by inhibiting the negative regulatory effects of miR‐130 on PPARγ and ultimately alleviating pulmonary hypertension.

## 4 DISCUSSION

PAH is a chronic progressive disease characterized by pulmonary vascular remodelling, ultimately leading to RVH and even death.^23^, ^24^, ^25^ Abnormal proliferation, migration and apoptotic resistance in PASMCs are central aspects of pulmonary vascular remodelling. The absence of specific drugs that directly target the vascular remodelling process has led to high end‐stage annual mortality in PAH.^26^, ^27^ Therefore, the search for potential drugs that modulate PASMCs has been a research hotspot in PAH in recent years. In this study, we provide new evidence that FGF21 inhibits hypoxia‐induced proliferation and migration of PASMCs, reverses PASMC resistance to apoptosis, and ultimately improves pulmonary vascular remodelling and alleviates pulmonary hypertension by downregulating the negative regulatory effect of miR‐130 on PPARγ.

FGF21 is a newly identified endogenous cardiovascular system protective factor with antagonistic effects on vascular smooth muscle cell proliferation.^28^, ^29^ Our previous study found that exogenous FGF21 upregulated the PPARγ expression, inhibited hypoxia‐induced PASMC proliferation, improved pulmonary artery remodelling and reduced pulmonary artery pressure,^10^, ^11^ suggesting that FGF21 may be a promising therapeutic agent for PAH. However, the associated genetic signalling mechanisms are unclear. There is increasing evidence that miRNAs are involved in the amelioration of cardiovascular injury by FGF21. Guo et al. reported that FGF21 prevents atherosclerosis by suppressing miR‐33 expression.^14^ Hu et al. found that FGF21 protected cardiomyocytes from I/R injury by increasing miR‐145 expression.^15^ Our previous study also found that FGF21 was able to inhibit inflammation in endothelial cells by regulating miR‐27b/PPARγ.^30^ However, the regulatory mechanism of FGF21 in PASMCs has not yet been reported. To explore the miR signalling mechanisms associated with the FGF21/PPARγ axis, we performed mRNA sequencing of lung tissues from FGF21‐treated PAH and hypoxic PAH mice. Forty‐five miRNAs were found to be significantly downregulated in the lung tissues of FGF21‐treated PAH mice. Bioinformatic analysis and literature review were used to search for miRNAs that may be involved in PASMC regulation. We identified the regulatory role of miR‐130 in the cardiovascular system, which has received increasing attention. Bertero et al. found that miR‐130/301 is involved in regulating apelin‐miR‐424/503‐FGF2 signalling in endothelial cells, and that miR‐130/301 regulates the STAT3‐miR‐204 signalling pathway to promote phenotypes associated with PAH in smooth muscle cells.^16^, ^17^, ^18^ PCR also confirmed a significant increase in miR‐130 in *in vitro* and *in vivo* hypoxia models, all of which suggest that miR‐130 may play an important role in the alleviation of PAH by FGF21, thus prompting our research interest. In this study, we used right heart catheterization to detect RVSP, cardiac ultrasound to detect PAAT and PAVTI to assess changes in pulmonary artery pressure levels, and H&E staining and immunofluorescence to reflect the extent of pulmonary vascular thickening. It was found that the miR‐130 agomir counteracted the beneficial effect of FGF21 on pulmonary artery pressure and vascular remodelling. PASMC migration was assessed using transwell and cell scratch assays, and PASMC proliferation was detected using CCK8, BrdU and western blotting. FGF21 was found to inhibit the promoting effect of the miR‐130 mimic on PASMC migration and proliferation. Finally, TUNEL immunofluorescence and western blotting results revealed that FGF21 was able to correct the apoptotic resistance caused by miR‐130 elevation. Above all, FGF21 regulates various phenotypic changes in PAH through miR‐130.

A recent report claimed that miR‐130 could exacerbate myocardial injury caused by acute myocardial infarction by targeting PPARγ.^31^ We also predicted that PPARγ is one of the direct targets of miR‐130 through bioinformatics analysis. This result is expected, as our studies^10^, ^11^ and those of others^22^ have confirmed that PPARγ is an important regulator of PAH development. PPARγ maintains lung health by regulating the cell cycle and proliferation.^32^ Idris‐Khodja et al. reported that PPARγ could enhance human PASMC apoptosis and protect against inflammation and oxidative stress.^20^, ^21^ Moreover, our previous study^10^, ^11^ revealed that PPARγ is one of the downstream pathways of FGF21. In this study, we confirmed the direct negative regulatory effect of miR‐130 on PPARγ using a dual luciferase reporter gene and miR‐130 up‐ and downregulation. The results showed that miR‐130 negatively regulated PPARγ protein levels by directly interacting with the 3'UTR of PPARγ in PASMCs. In contrast, the miR‐130 inhibitor failed to inhibit hypoxia‐induced PASMC proliferation, migration and apoptotic resistance when co‐transfected with siPPARγ, suggesting that the miR‐130/PPARγ regulatory axis is an important link in the development of hypoxia‐induced PAH. Furthermore, miR‐130 was found to reverse the upregulation of PPARγ by FGF21. Our data suggest that miR‐130 is an important mediator of the FGF21/PPARγ axis in the development of PAH.

This study also has certain shortcomings, for example, the relationship between miR‐130 and PPARγ was predicted based on previous experimental findings and the bioinformatics approach. Although the association was illustrated to some extent, direct evidence needs to be obtained by constructing lung‐specific PPARγ‐knockout mice. In addition, the exact role of the FGF21/miR‐130/PPARγ axis in PAH needs to be further validated in combination with clinical samples.

## 5 CONCLUSIONS

**FIGURE 8:**
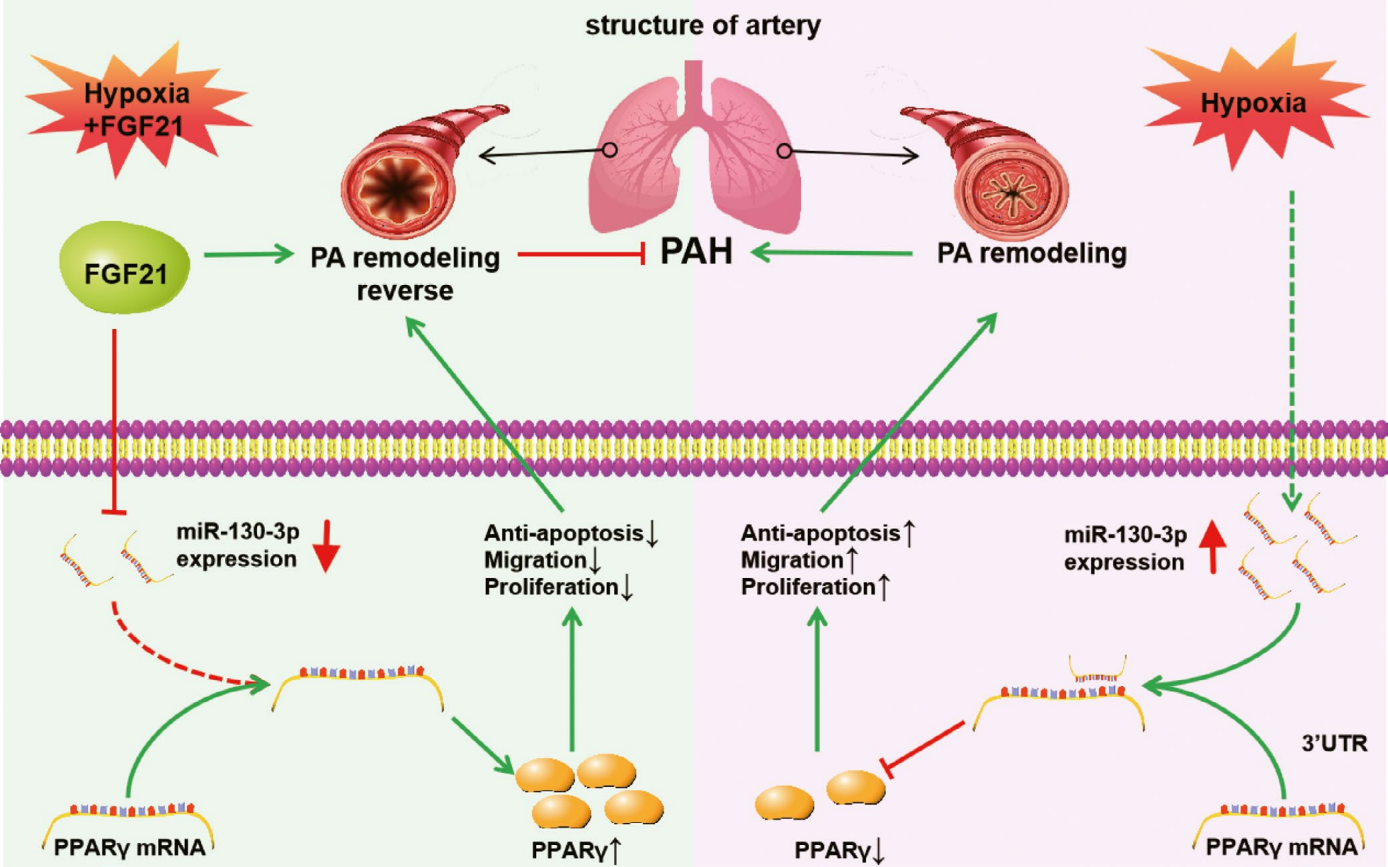
Signalling pathways in this experiment. FGF21 exerted protective effects against hypoxic PASMCs by inhibiting the negative regulatory effects of miR‐130 on PPARγ

This study identified a new mechanism, by which FGF21 attenuates hypoxia‐induced PAH by inhibiting the negative regulation of miR‐130 on PPARγ (Figure 8), further enriching the understanding of the regulatory molecular mechanism of FGF21 in PAH. This provides a new idea for epigenetics‐based PAH treatment.

## CONFLICT OF INTEREST

The authors have declared that no competing interest exists.

## AUTHOR CONTRIBUTION

**Meibin Wang:** Conceptualization (equal); Data curation (equal); Formal analysis (equal); Methodology (equal); Writing – original draft (equal). **Lihuang Su:** Conceptualization (equal); Data curation (equal); Formal analysis (equal); Methodology (equal); Project administration (equal); Resources (equal); Writing – original draft (equal). **Junwei Sun:** Software (supporting); Validation (supporting). **Luqiong Cai:** Data curation (supporting); Formal analysis (supporting); Investigation (supporting). **Xiuchun Li:** Investigation (supporting); Methodology (supporting); Software (supporting). **Xiayan Zhu:** Investigation (supporting). **Lanlan Song:** Investigation (supporting). **Jingyin Li:** Investigation (supporting). **Shuolan Tong:** Investigation (supporting). **Qinlian He:** Data curation (supporting); Investigation (supporting); Validation (supporting). **Mengsi Cai:** Formal analysis (supporting); Investigation (supporting). **Lehe Yang:** Methodology (supporting); Project administration (supporting); Writing – review & editing (supporting). **Yanfan Chen:** Conceptualization (supporting); Writing – review & editing (supporting). **Liangxing Wang:** Conceptualization (supporting); Methodology (supporting); Project administration (supporting); Writing – original draft (supporting); Writing – review & editing (supporting). **Xiaoying Huang:** Conceptualization (supporting); Funding acquisition (lead); Methodology (supporting); Project administration (supporting); Supervision (lead); Writing – original draft (supporting); Writing – review & editing (lead).

## Supporting information

Supplementary Material

## Data Availability

All relevant data and materials are stored at the Key Laboratory of Heart and Lung of Wenzhou Medical University and can be obtained from the first author and corresponding author.
