# Supplementary material for: FGF21 attenuates pulmonary arterial hypertension via downregulation of miR‐130, which targets PPARγ
Source: J Cell Mol Med. 2022 Jan 6;26(4):1034–49. doi: 10.1111/jcmm.17154 (PMC8831951; doi:10.1111/jcmm.17154)
Supplement: Supplementary file 1 — Supplementary Material [file JCMM-26-1034-s001.docx]

**Fibroblast growth factor 21 attenuates pulmonary arterial hypertension via** **downregulation of miR-130, which targets peroxisome proliferator-activated receptor gamma**

Meibin Wang^1†^, Lihuang Su^1†^, Junwei Sun^1^, Luqiong Cai^1^, Xiuchun Li^1^, Xiayan Zhu^1^, Lanlan Song^1^, Jingyin Li^1^, Shuolan Tong^1^, Qinlian He^1^, Mengsi Cai^1^, Lehe Yang^1^, Yanfan Chen^1^, Liangxing Wang^1^*, Xiaoying Huang^1^*

^1^Division of Pulmonary Medicine, The First Affiliated Hospital of Wenzhou Medical University, Key Laboratory of Heart and Lung, Wenzhou, Zhejiang, 325000, China

^†^These authors contribute equally to this work.

*Correspondence to: Xiaoying Huang, E-mail: huangxiaoying@wzhospital.cn; Liangxing Wang, E-mail: wzyxywlx@163.com

**Supplemental data**

**Figure Supplement 1 PPARγ activation inhibited hypoxia-induced PASMC proliferation**

**
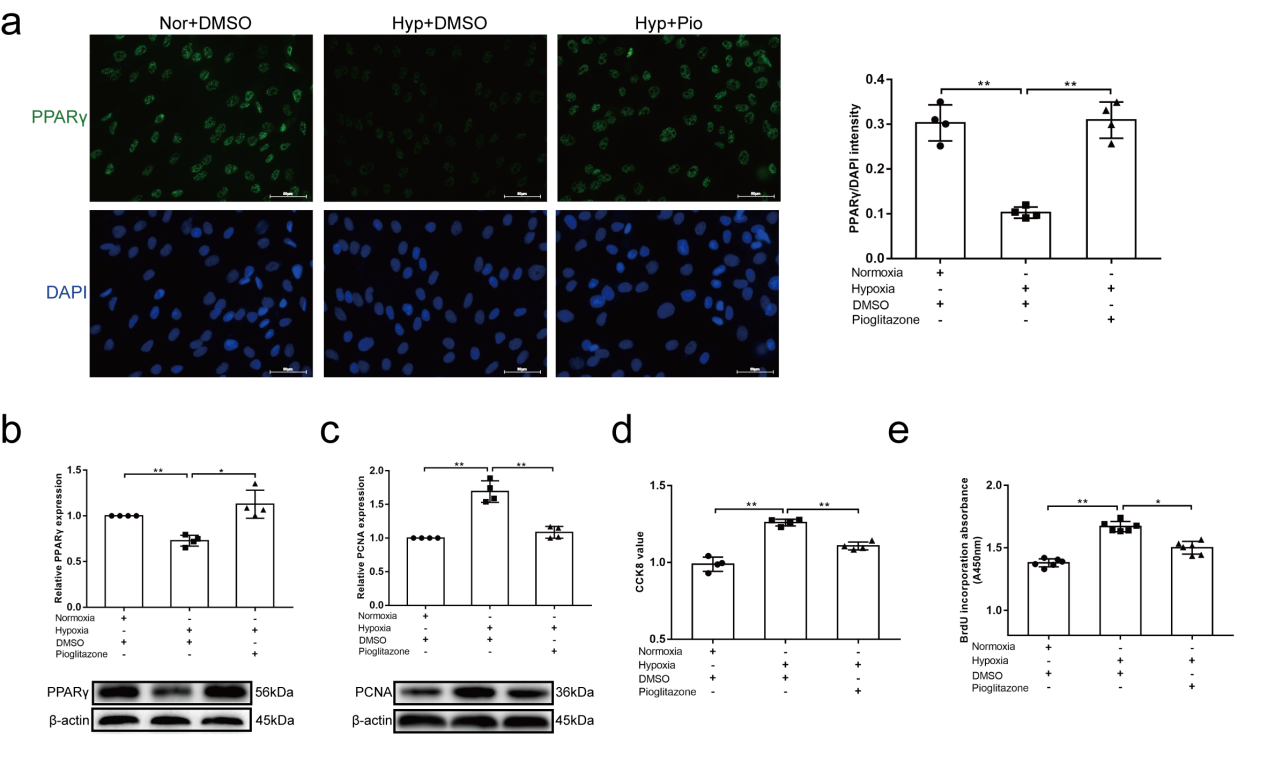
**

1. Immunofluorescent staining for PPARγ (green) and DAPI (nuclear DNA; in blue). The histogram represents the quantification of the ratio of PPARγ to DAPI intensity (n = 4) (×100; scale bars indicate 200 µm). (b) Western blotting for PPARγ and (c) PCNA in cultured PASMCs (n = 4), β-actin was used as a loading control. (d) CCK-8 assay (n = 4) and (e) BrdU incorporation study (n = 6) were used to evaluate cell viability. Data are presented as the mean ± SD. Single comparisons and multiple comparisons were analyzed with Student’s t-test and one-way ANOVA, respectively. **p*<0.05, ***p*<0.01.

**Figure Supplement 2 PPARγ activation inhibited hypoxia-induced PASMC migration**

**
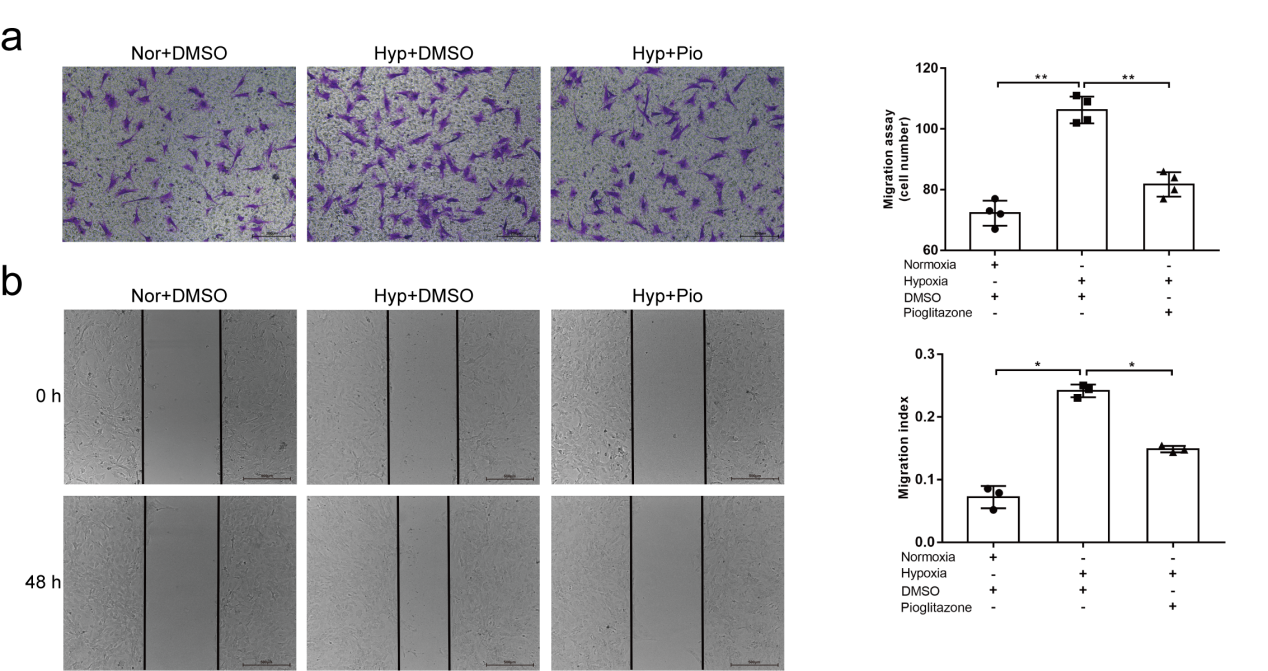
**

Cell migration was determined by (a) Transwell assays at 24 h (n = 4) (×100; scale bars indicate 200 µm) and (b) wound-healing assays at 0 h and 48 h (n = 3) (×40; scale bars indicate 500 µm). Data are presented as the mean ± SD. Single comparisons and multiple comparisons were analyzed with Student’s t-test and one-way ANOVA, respectively. **p*<0.05, ***p*<0.01.

**Figure Supplement 3 PPARγ activation promoted hypoxia-induced PASMC apoptosis**

**
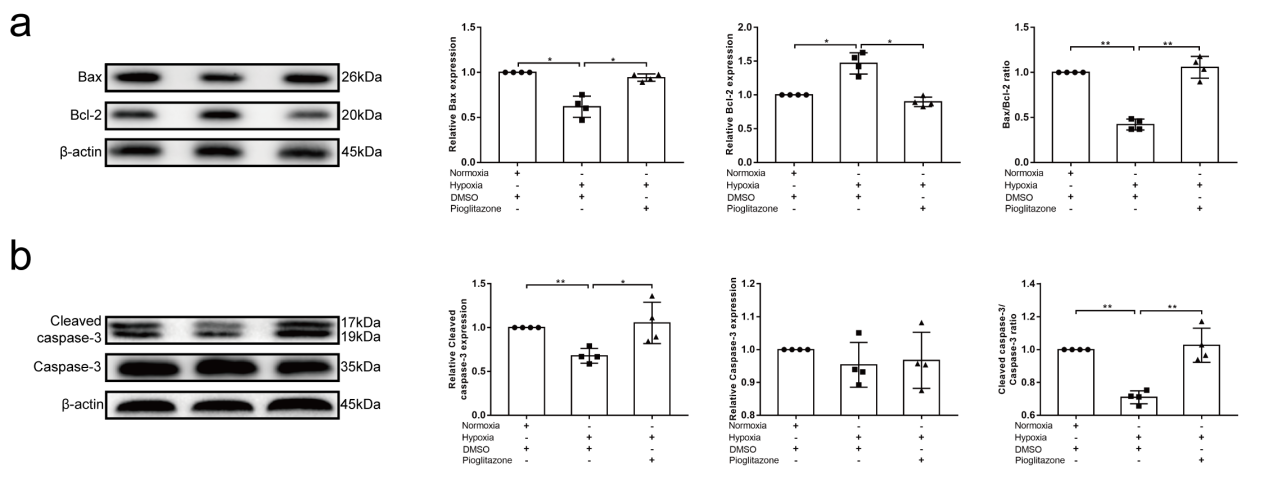
**

(a) Western blotting for Bax and Bcl-2 in cultured PASMCs (n = 4). (b) Western blotting for cleaved caspase-3 and caspase-3 in cultured PASMCs (n = 4). β-actin was used as a loading control. Data are presented as the mean ± SD. Single comparisons and multiple comparisons were analyzed with Student’s t-test and one-way ANOVA, respectively. **p*<0.05, ***p* <0.01.

**Figure Supplement 4 Efficiency of miR-130 inhibitor**

**
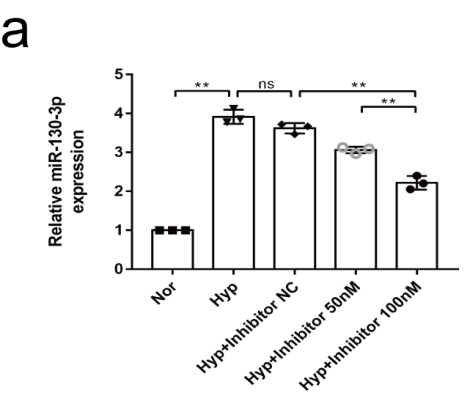
**

(a) qRT-PCR was used to detect the efficiency of miR-130 inhibitor (n = 3). The miRNA level is normalized to U6 by the 2^−ΔCt^ method before comparative analysis. Data are presented as the mean ± SD. Single comparisons and multiple comparisons were analyzed with Student’s t-test and one-way ANOVA, respectively. **p*<0.05, ***p*<0.01.

**Figure Supplement 5 The siRNA knockdown efficiency of PPARγ**

**
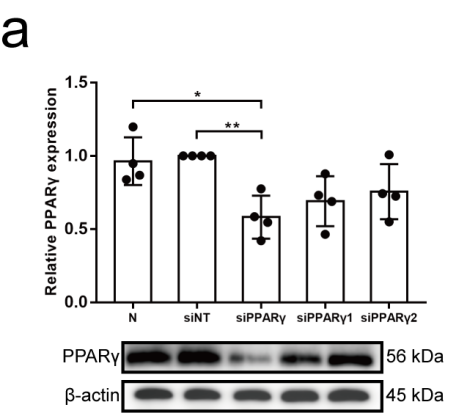
**

(a) The siRNA knockdown efficiency of PPARγ was detected by western blotting (n = 4). β-actin was used as a loading control. Data are presented as the mean ± SD. Single comparisons and multiple comparisons were analyzed with Student’s t-test and one-way ANOVA, respectively. **p*<0.05, ***p*<0.01.

**Figure Supplement 6 FGF21 reduces the proliferation of hypoxia-induced PASMCs by inhibiting the negative regulatory effects of miR-130 on PPARγ**


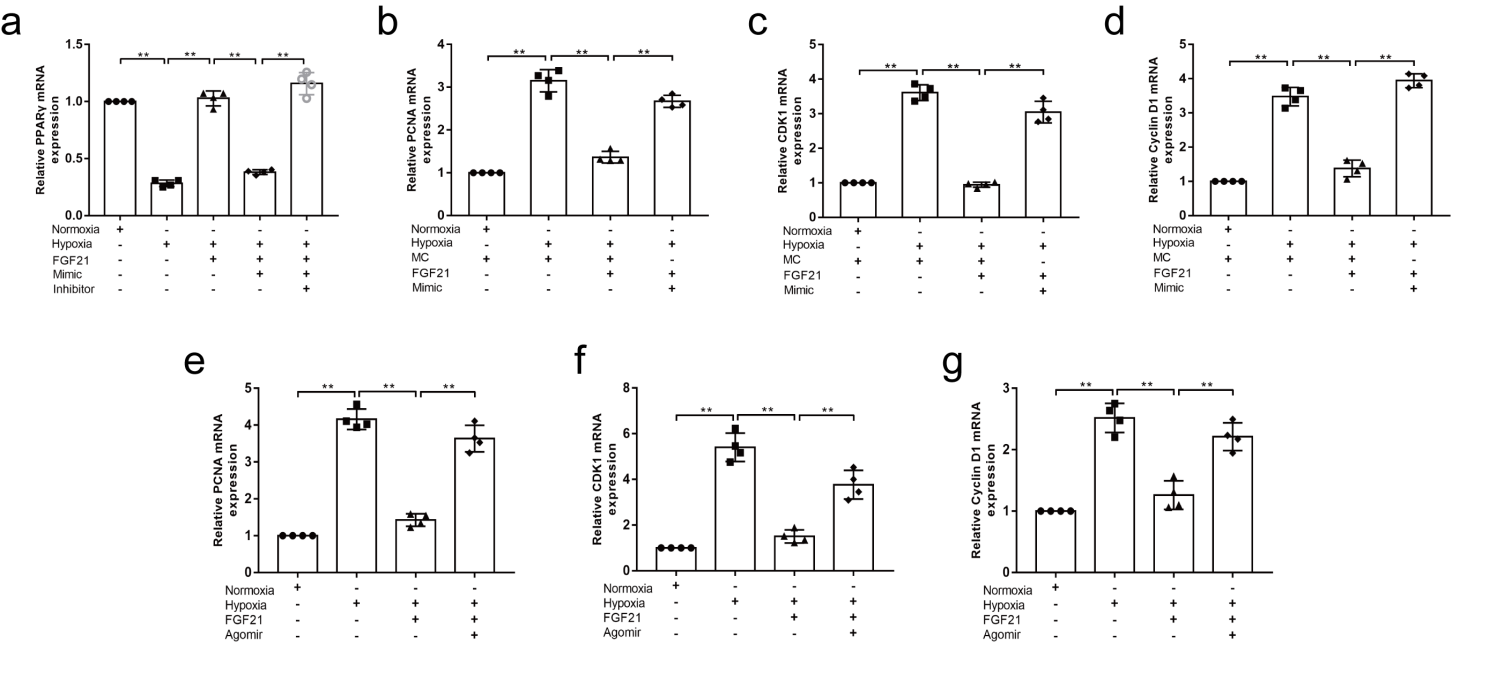


(a) qRT-PCR was used to detect the expression of PPARγ in PASMCs treated with FGF21, miR-130 mimic or miR-130 inhibitor. The mRNA level is normalized to β-actin by the 2^−ΔCt^ method before comparative analysis (n = 4). (b-d) qRT-PCR was used to detect the expression of PCNA, CDK1 and cyclin D1 in PASMCs treated with FGF21 or miR-130 mimic. The mRNA level is normalized to β-actin by the 2^−ΔCt^ method before comparative analysis (n = 4). (e-g) qRT-PCR was used to detect the expression of PCNA, CDK1 and cyclin D1 in mice lung tissue. The mRNA level is normalized to β-actin by the 2^−ΔCt^ method before comparative analysis (n = 4). Data are presented as the mean ± SD. Single comparisons and multiple comparisons were analyzed with Student’s t-test and one-way ANOVA, respectively. **p*<0.05, ***p*<0.01.

**Figure Supplement 7 Efficiency of exogenous miR-130 agomir**

**
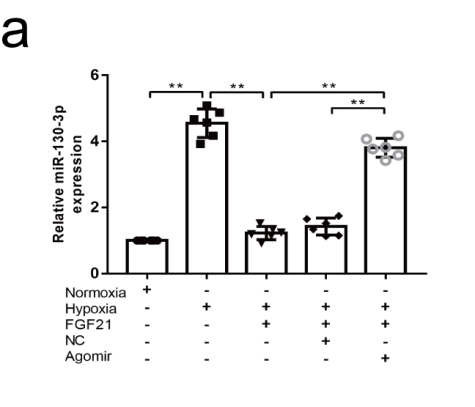
**

(a) qRT-PCR was used to detect the efficiency of miR-130 agomir. The miRNA level is normalized to U6 by the 2^−ΔCt^ method before comparative analysis (n = 6). Data are presented as the mean ± SD. Single comparisons and multiple comparisons were analyzed with Student’s t-test and one-way ANOVA, respectively. **p*<0.05, ***p*<0.01.

**Table Supplement 1 Primer sequences of miRNA used for qRT-PCR analyses**

| Gene name | Forward primer | Reverse primer | RT primer |
| --- | --- | --- | --- |
| miR-130 | GCGCAGTGCAATGATGAAA | AGTGCAGGGTCCGAGGTATT | GTCGTATCCAGTGCAGGGTCCGAGGTATTCGCACTGGATACGACATGCCC |
| U6 | AGAGAAGATTAGCATGGCCCCTG | ATCCAGTGCAGGGTCCGAGG | GTCGTATCCAGTGCAGGGTCCGAGGTATTCGCACTGGATACGACAAAATA |

**Table Supplement 2 Primer sequences of mRNA used for qRT-PCR analyses**

| Gene name | Forward primer | Reverse primer |
| --- | --- | --- |
| PPARγ | CTCTGTGGACCTCTCCGTGAT | GAATGGAATGTCTTCATAGTGTGG |
| PPARγ 3'UTR | CCGCTGACAACGTGTTCCT | TTCTTAGGTGTCAGATTTTTTTCCC |
| PCNA | AGAGGAGGCGGTAACCATAGA | GGAGACAGTGGAGTGGCTTTT |
| CDK1 | GCCAGAGCGTTTGGAATACC | GGAGTGGAGTAACGAGCCGA |
| Cyclin D1 | GGATCTGTCCTGCAAAGTTGAAG | TCCAGGTAATGCCATCATGGT |
| β-actin | TCAAGATCATTGCTCCTCCTGAG | ACATCTGCTGGAAGGTGGACA |
